# Factors Related to Sports Participation in Brazil: An Analysis Based on the 2015 National Household Survey

**DOI:** 10.3390/ijerph17176011

**Published:** 2020-08-19

**Authors:** Felipe Magno, Carla Schwengber ten Caten, Alberto Reinaldo Reppold Filho, Aline Marian Callegaro, Alan de Carvalho Dias Ferreira

**Affiliations:** 1 Graduate Program on Industrial Engineering, Federal University of Rio Grande do Sul, Porto Alegre 90035-190, Brazil; carlacaten@gmail.com; 2 School of Physical Education, Physical Therapy and Dance from the Federal University of Rio Grande do Sul; Porto Alegre 90690-200, Brazil; alberto.reppold@ufrgs.br (A.R.R.F.); 3105.ferreira@gmail.com (A.d.C.D.F.); 3 Interdisciplinary Department of Campus Litoral Norte from the Federal University of Rio Grande do Sul; Tramandaí 95590-000, Brazil; nimacall@gmail.com

**Keywords:** sports participation, physical activity, health, public policies, logistic regression

## Abstract

The academic interest in analyzing the correlates of sports participation in several countries has increased recently. Nevertheless, in developing countries, which do not monitor sportive data, this type of investigation is still scarce. This study aims to analyze socioeconomic, motivational, and supportive factors related to sports participation in Brazil. Data from the 2015 National Household Survey—Supplementary Questionnaire of Sports and Physical Activities are examined. In the survey, 71,142 individuals older than 15 years were interviewed (mean age 43.12 years; 53.83% women and 46.17% men). Logistic regression is used for analyzing the data. Results demonstrate a low participation in sports (23.38%). Sports participation declines with increasing age (2% less per year), increases with higher educational level (graduated 5.9 times more), and males prevail in the sporting context (2.3 times more). The main obstacle to women’s participation is the lack of sports facilities, and for men the lack of time and health problems. Men practice sports mainly due to socialization, fun, and competition, and women due to medical recommendation. Soccer was the most practiced sport (28.1%), predominating among men. Public policies on sports promotion for fun and socialization may increase male participation, and investments in sports facilities may increase female participation.

## 1. Introduction

The policies of various governments direct efforts to promote sports participation in a population. According to Nicholson, Hoye, and Houlihan, the main reason for the governmental interest in sports participation is its potential contribution to the improvement of several health problems [1], as well as the reduction of the costs associated with them [2]. Recent studies show that even low levels of physical activity can reduce mortality or promote health improvements [3,4,5]. Moreover, sports and physical activity not only contribute to physical fitness, but also have an impact on mental, emotional, and social well-being of adults, adolescents, and children [6,7]. It is worth mentioning that there is an intersection between physical activity and sport, and a clear distinction is necessary. Physical activity is understood as any bodily movement executed for domestic work, occupational labor, transportation, sport, exercise, leisure, or recreation. Sport is understood as subset of physical activities in which the participants have common expectations and objectives, performed individually or collectively [8].

Despite its relevance, sports participation has demonstrated low rates in several countries. An analysis clustered by regions of Europe identified that in many regions from Italy, France, Portugal, Greece, Poland, Romania, Bulgaria, Czech Republic, and Slovakia, and in some regions from Belgium, Austria, and the United Kingdom, only 11.6% and 17.4% of the people practiced sports and physical activity at least three times per week, respectively [9]. Additionally, a study that analyzed global physical activity levels found that approximately 35% of the population in Europe were physically inactive. When compared with Europe, the American continent achieved even more alarming rates, where about 43% of the population was considered physically inactive [10]. A survey conducted in three South American countries compared the practice of physical activity among Colombia, Mexico, and Brazil. The results demonstrated that Brazil had the lowest percentage of moderate-to-vigorous-intensity leisure of physical activity (12.6% versus 21.6% in Colombia and 20.4% in Mexico) [11].

Previous researches have mainly examined the influence of socioeconomic factors (age, gender, income, educational level, etc.) in sports participation [12,13,14,15,16,17]. In addition, different groups of motives for why people get involved in sports, such as health and fitness, enjoyment and recreation, relaxation, appearance, socialization, and competition, have also been identified [18,19]. Other authors linked availability of sport infrastructure [20,21] and legacies of Olympic Games to an increase in sports participation [22,23,24]. Downward, Lera-López, and Rasciute investigated socioeconomic, lifestyle and motivational factors related to sports participation, as well as availability of sports facilities and governmental support. The results showed that the decision to take part in sports and the frequency of sports participation of men and women are affected by different factors. For example, difficulties to access sports facilities, low income, or household activity negatively impacts sport participation among women, and alcohol consumption was associated to a reduction in the likelihood of participation for males [25]. Therefore, public policies that consider the wide range of factors related to sports participation may bring positive results.

Different approaches are employed to identify the factors related to sports participation. The present study aims to address most of the factors presented above in the context of Brazil, a developing country which hosted the Olympics in 2016 and adopted a series of investments aimed at the improvement of national sports [26]. One of the measures taken by the country’s Ministry of Sport was, in partnership with the Brazilian Institute of Geography and Statistics (Instituto Brasileiro de Geografia e Estatística—IBGE, in Portuguese), the conducting of a survey that aimed at describing the profile of the (non-) participant in sports in Brazil [27]. In the 2015 National Household Survey (Pesquisa Nacional por Amostra de Domicílio—PNAD, in Portuguese)—Supplementary Questionnaire of Sports and Physical Activities, 71,142 individuals older than 15 years were interviewed about motivational factors to practice sports or not, and supportive factors (professional guidance, participation in competition, and place of practice) related to the practice of different sports [27]. It is important to explain that the term “sport” in the context of this research included simple forms of physical activity, such as walking. This occurred because the interviewees were free to mention the main sport practiced without a predetermined list, generating broader responses, similar to other studies that also used self-reported measures [28,29].

The Brazilian PNAD has been realized for forty-nine years, aiming to evaluate the socioeconomic development of the country. In the edition of 2008, an analysis of physical activity in leisure time and during transportation from and to work was added to the research, but sports participation was not examined. The results showed that one in five Brazilians did not practice any physical activity [30]. The edition of 2015 was the first-time sport participation was included in PNAD, which apparently demonstrated the increase of the Brazilian government interest. However, in 2016, the special annual edition of PNAD was interrupted. The Supplementary Questionnaire of Sports and Physical Activities from PNAD 2015 was the first and the last broader survey to examine sports participation in the country.

The present study aims to analyze, based on data of the survey above, the socioeconomic, motivational, and supportive factors related to sports participation in Brazil. For that purpose, prevalence of practice, motives for practicing and not practicing sports, and main sports practiced were analyzed, stratified by age, gender, professional guidance, participation in competition, and place of practice.

## 2. Methodological Procedures

The PNAD is a household sampling research system carried out in the 26 Brazilian states and the Federal District. It investigates various socioeconomic and demographic characteristics of the Brazilian population. In the 2015 edition of PNAD, 151,189 households were selected, after the probabilistic sampling composed of three stages: cities, census sectors, and households’ unities. Among these, 125,034 households were occupied and 94.3% answered the survey. For the supplement “Practices of Sports and Physical Activities”, 94,814 households were selected by simple random sampling, considering those already sampled for the PNAD 2015. Among the selected households, 82.8% of the households were occupied. From the occupied residences, the response rate was 90.7%, totalizing 71,142 households. From each household in the sample, a person of 15 years old or more was selected, also with equal likelihood, to answer the questionnaire [27]. Raw data and codifications of PNAD can be found in the IBGE website [31]. A summary table (Table 1) of the sample is presented, classified by gender, age groups, and educational level.

In the supplement “Practices of Sports and Physical Activities” of 2015, the surveyed participants were questioned: whether they practiced any sport (yes/no); the main motive for practicing or not sport (one), the main sport practiced (one), the place of practice (public space/free sports facilities/paid sports facilities), whether they had professional guidance (yes/no), and whether they participated in competition (yes/no). Every question alluded to the 365-day reference period.

Response variables analyzed were practice or not sport, motive for practicing sports, motive for not practicing sports, the main sport practiced in the period. All were stratified by gender, age, and educational level predictors. Regarding the main sport practiced, the following were also analyzed: the presence of professional guidance, place of practice, and participation in competition. For analytical purposes, the sports mentioned were distributed into 22 categories, named “types of sports” (Appendix A Table A1).

As it was assumed that the relationship between the variables was of dependence, and by the fact that most of them, including outcome variables, are categorical, a logistic regression (binary and nominal) was chosen. According to Kleinbaum and Klein, this method allows the use of a regression model to calculate or predict the probability of a specific event occurring based on the odds ratio [32]. For continuous predictors, odds ratios greater than 1 indicate that the comparison outcome is more likely than the reference outcome as the predictor increases, and odds ratios less than 1 indicate that the reference outcome is more likely than the comparison outcome. For categorical predictors, odds ratios greater than 1 indicate that the comparison outcome becomes more likely relative to the reference outcome and odds ratios less than 1 indicate that the comparison outcome becomes less likely relative to the reference outcome. The confidence interval (CI) used was 95%. Confidence intervals are ranges of values that are likely to contain the true values of the odds ratios and use the normal distribution. The *p*-value of statistical significance assumed was *p* ≤ 0.05. The statistical analysis was performed in the Software Minitab 18.1 (Minitab, LLC, State College, PA, United States).

For the binary logistic regression, the “deviance goodness-of-fit test” was used. If the *p*-value is lower than the significance level, it is possible to determine that the model does not fit the data. For the nominal logistic regression, the “test that all slopes are zero” was used. If the *p*-value is less than or equal to the significance level, it is possible to determine that there is a statistically significant association between the response variable and at least one of the predictors.

To analyze the practice or not of sports, a binary logistic regression was conducted considering the dependent variable as follows: did not practice sports = 0; practiced sports = 1. As predictor variables, gender, age, and educational level were considered (consult Table 2).

Regarding the individuals who answered not practicing any sports, a nominal logistic regression was performed to assess the motives for not practicing sports. The selected outcome variable was “motive for not practicing sports”, divided into the following categories: does not like, lack of time, health problems, lack of sports facilities, lack of company, lack of money. “Lack of sports facilities” was used as reference level, as it is the motive that may receive more influence from public policies. Age, gender, and educational level were considered as predictor variables. For the educational level, only the comparisons between “Graduate” and the level of reference “Unschooled” are presented to facilitate data interpretation (consult Table 3).

Considering the individuals who have answered practicing sports, a nominal logistical regression was performed. The selected outcome variable was “motive for practicing sports”, divided into the following categories: medical recommendation, socialization, life quality, fun, fitness, competition. The category “medical recommendation” was used as the reference level, as it is the motive that receives more external influence, depending less on the individual’s conscientiousness of the relevance of practicing sports. Age, gender, and educational level were considered as predictor variables. For the variable “educational level” only comparisons between the “graduate” and the reference level “unschooled” are presented, to facilitate data interpretation (consult Table 4).

Additionally, the main type of sports practiced in Brazil were described (consult Table 5). To identify variables that impact the choice for practicing certain types of sports, a nominal logistic regression was performed. Thus, “main sport practice” was selected as the outcome variable. “Soccer” was used as the reference level, as it is considered the national sport, and can be a good parameter to observe the nuances of other sports disciplines in the country. The predictor variables were age, gender, presence of professional guidance, participation in competition, and place of practice. In relation to the place of practice, the category “public space” was used as the reference level, aiming to demonstrate the impact that sport facilities (paid or free) have in the practice of certain sports (consult Table 6).

## 3. Results

In the PNAD, of the 71,142 people qualified to answer the survey, 16,630 (23.38%) noted practicing sport in the reference period. Table 2 presents a binary logistic regression analyzing the choice of practicing sports, by age, gender, and educational level.

The results of the Table 2 indicate that for the variables analyzed, there is a statistically significant association with the outcome variable (*p* ≤ 0.05). Regarding age, the result shows that for each additional year, the odds of a person practicing sports decreases by approximately 2%. Concerning gender, the data show that men are around twice as likely to practice sports than women. Regarding educational level, by observing the extreme categories, it is noted that there is about a six times greater chance of an individual with a graduate degree practicing sport compared with those without education (unschooled). The result of the “deviance goodness-of-fit test” was 0.19.

From the 71,142 interviewees, 54,512 said they had not practiced any sport in the reference period. The possible motives were lack of time (37.72%), does not like (34.72%), health problems (20.23%), lack of sports facilities (2.63%), lack of money (1.76%), lack of company (1.74%), and other motives (1.49%). Table 3 presents a logistic regression analyzing the motives for not practicing sports, by age, gender, and educational level. The category “other motives” was excluded from the analysis, reducing the sample to 53,699 people.

Table 3 shows that in almost all comparisons, there is a statistically significant association with age and gender (*p* ≤ 0.05). However, regarding the educational level variable, only the first comparison presents a statistically significant result. In this case, for example, the results indicate that individuals with a graduate degree are three times more likely to mention the lack of time as an obstacle to participating in sports than individuals with no education, when compared with the reference level (lack of sports facilities). The result of the “test that all slopes are zero” was 0.00.

From the 71,142 interviewees, 16,630 said they practiced sport in the reference period. The possible motives were life quality (28.07%), fun (28.01%), fitness (20.36%), medical recommendation (11.05%), competition (8.77%), socialization (3.75%), and other motives (0.43%). Table 4 presents a logistic regression analyzing the motives for practicing sports, by age, gender, and educational level. The category “other motives” was excluded from the analysis, reducing the sample to 16,549 people.

Table 4 shows that all comparisons with age and gender are statistically significant. However, only the second and fourth association with educational level presented statistically significant results. In this case, for example, the results indicate that individuals with a graduate degree are three times more likely to mention life quality as a motive to participate in sports than individuals with no education, considering the reference level (medical recommendation). The result of the “test that all slopes are zero” was 0.00.

Furthermore, respondents who reported practicing sports were instructed to indicate which was the main sport practiced in the period. Table 5 shows the distribution of these sports by category.

Considering the main sports practiced, a logistic regression was performed by age, gender, presence of professional guidance, participation in competition, and place of practice. To ensure the quality and reliability of the results, a minimum of a hundred (100) events was required. Hence, nine types of sports (small balls and rackets, skateboard and skating, water sports, basketball, handball, sports with animals, adventure sports, car sports, cards and board games) were removed. The category “others” was also removed from the analysis, which reduced the sample to 15,319 people. The main results are presented in Table 6.

Table 6 shows that most of the comparisons are statistically significant. In relation to the variable gender, women are more likely to practice all other sports examined than soccer. For example, women are around 97 times more likely to practice dance and ballet. The presence of professional guidance is more likely to occur in any other sport than soccer. For example, combat and martial arts are around 140 times more likely to be monitored by a professional. Participation in competition is less likely to occur in most of the other sports when compared with soccer. Regarding the place of practice, sports such as cycling, walking, and athletics are less likely to depend on sports facilities than soccer. The result of the “test that all slopes are zero” was 0.00.

## 4. Discussion

The analyzed PNAD data show, initially, a low number of people that declared practicing sports. By analyzing the binary logistic regression executed, we found similar results to previous studies. The results indicate that older age has a negative relation with sports participation [15,16,33]. Men are more likely to practice sports than women [13,16]. Higher educational level is directly related to the increase in sport participation rate [34,35,36]. It is also known that people with higher education are more likely to have higher incomes [37], social support, and greater capacity to seek, understand, and act on health messages that promote sport and physical activity [38]. The data presented in the article indicate, for example, that the odds of someone with a graduate degree practicing sports is six times higher than the odds of individuals with no education.

When analyzing the reasons for not practicing sports, the nominal regression analysis indicated that increasing age generates higher chances of mentioning “health problems” as the main reason for not practicing sports. This reinforces the fact that with older ages, people have more chances of not practicing due to their medical condition. Furthermore, women are more likely to report the lack of sports facilities and the lack of money as major obstacles to sports practice. This result reinforces some studies that have already identified this relation (for example, [25]) and indicates that policies of investment in sports facilities or the availability of free access facilities, as well as use of idle spaces, can increase female participation in sports. Despite the importance of sport facilities in predicting participation, its influence depends on the type of sport and facility [21]. As for the educational level, the main impact is found in the comparison of the “lack of time” with the reference level, pointing out that the higher the educational level, the greater the chances of missing time for the practice of sports. Lack of time has been pointed out before as one of the main reasons for not practicing sports, by married people [39,40] or by those with larger family size [41]. Some analysis also showed that labor has a negative relation with sports participation (for example, [42]). Nonetheless, studies that specifically analyze this condition have not been found; however, it is possible to speculate that people with higher education have greater awareness of the importance of practicing sports, and that lack of time is reported as the biggest obstacle for performing these activities.

When analyzing the reasons for practicing sports, the nominal regression analysis indicated that all comparisons pointed to “medical recommendation” as a motive to practice sport as one grows older. Once more, the results follow the same interpretative logic. Older people are less likely to practice sports. Non-practitioners are more likely to mention “health problems” as the main reason for not participating. Participants, however, are more likely to do it because of a medical recommendation. The promotion of sport for the elderly can be considered an important public health policy. In a Brazilian study carried out with 679 men and women, a higher percentage of survival was found among adult practitioners of sports with moderate and vigorous intensity and with at least four months of previous involvement [43]. Still, the elderly with a higher score in the sports/gym domain had better scores in functional capacity [44].

In relation to the gender variable, the most relevant results show that men are about eight times more likely to practice sports looking for socialization and fun opportunities. This result is close to that described by Downward et al., which stated that men engage in sports to socialize [25]. The sport’s social context was also identified as a mechanism that helps men with a lower socioeconomic level to overcome isolation [45]. Moreover, men are nine times more likely to report “competition” as the main reason to engage in sports. This result aligns the vision that men are more likely to participate in more intense competitive sports, such as team sports; however, the ability to sustain this activity decreases with age [25]. On the other hand, women’s activities, which are usually associated with maintaining shape, are more sustainable throughout life (see, for example [46]). Regarding educational level, the most significant results show that people with a graduate degree are more likely to mention as reasons for participating “quality of life” and “physical fitness”. This result may be an indication that the educational level amplifies the perception of the importance of sport activities in life. Indirectly, a higher educational level may be associated to a higher income and, consequently, more available resources to perform physical activities [37].

Concerning the main sport practices, soccer and walking stand out, accounting for over 50% of the total. In a global study, walking has already been identified as the most popular practice in the Americas [29]. In addition, the promotion of walking has been reported as a viable public health strategy due to its popularity [47], and associated health benefits [48,49]. In relation to soccer, its popularity can be partly attributed to the fact that this sport is a tradition in many countries [29], especially in Brazil. It is noteworthy that soccer can provide more substantial benefits in aerobic conditioning, cardiovascular function, and reduced adiposity, compared with many other physical activities [50].

The nominal logistical regression aimed at analyzing the factors that influence the choice of certain sports. It is worth noting the increase in prevalence of sports such as “walking” and “swimming and diving” with advancing age. This can be explained by the fact that water sports and walking are considered by the population as low-impact and low-injury-risk activities. Moreover, this kind of activity is usually recommended by health professionals, reinforcing once more the results found and already mentioned. Nevertheless, global participation rates reflected a consistent pattern of participation in swimming, running, and walking throughout life, even at older ages [29], which may also be related to the results found. As for gender, the results show that women are more likely to practice all other kinds of sports when compared with soccer. This result demonstrates that Brazil is not a soccer country, but rather a men’s soccer country. This factor reinforce socialization as a motivational factor for men to pursuit sport. It is known that team sports are social in nature and people are inherently motivated to participate in sport due to this aspect [51,52]. Participation in team sports, in addition to producing physical health benefits, can improve psychological and social health [6]. As men predominate in soccer, and represent a large part of Brazil’s context, it is expected that socialization is highlighted as a motivational element. Women, for example, are about 97 times more likely to practice dance and ballet than soccer. However, in the women’s context, soccer is also behind sports such as swimming, volleyball, combat sports and martial arts, track and field, and cycling.

Furthermore, some support factors for the practice of certain sports have been evaluated. The results indicate that the presence of “professional guidance” is stronger in all other kinds of sports than in soccer. The most prominent type of sport in this case was “combat sports and martial arts”. It is possible to conjecture that this type of sport has a strong connection with the teacher/master aspect, which combines technical expertise and discipline attached to this kind of practice. Considering the “participation in competition or not”, for most types of sports the comparison with soccer was negative. The exception is the category “athletics”, in which there is a 60% higher probability of participating in competitions than in soccer. One circumstance that can justify this finding is the perception that the term “athletics”, as opposed to other activities, is directly associated with high performance. As for “place of practice”, the results show that sports facilities have a lower impact on participation rates in sports such as cycling, athletics, and activities like walking. It is known that access to facilities can inhibit or facilitate participation in physical activities [53,54,55,56], as can the cost of associated equipment [35,57]. Thus, activities such as walking, running, and cycling may present opportunities for practices that require simpler motor skills, low equipment costs, and non-mandatory specific sports facilities. In the study, sports such as volleyball and futsal are more likely to be held in sports facilities than soccer, however, there are no big differences between practicing in free or paid facilities. This may indicate the greater provision of courts and gymnasiums in the country. Activities such as “bodybuilding/weightlifting”, “fitness sports”, “dance/ballet”, “gymnastics”, “combat sports and martial arts”, and “swimming/diving” are mainly held in paid facilities. However, the costs incurred in club membership fees, equipment, and transportation may limit these options of activities to individuals economically disfavored [55,58,59]. Besides, as mentioned, these activities are mainly performed by women, which leads us to assume that access to sports facilities is the biggest obstacle for women in sport.

Participating in some sports can require specialized facilities and orientation, and the higher the gross domestic product per capita in a country, the higher the likelihood of an individual having access to it. Besides, in a comparison of 11 countries, the availability of low-cost facilities was less likely to be reported in Brazil and Colombia, and more likely in Canada and New Zealand. It was also reported that access to low-cost facilities can significantly impact the levels of practice [60]. In a study carried out with approximately 700 people in the city of Curitiba, Brazil, it was reported that the proximity and the amount of public leisure spaces were associated with higher levels of moderate-to-vigorous physical activity in adults [28]. Moreover, studies have reported less facilities in poorer neighborhoods when compared with wealthier ones, indicating that the environment hinders the inclusion of the underprivileged in sports [61]. In addition, it was found that neighborhoods with low socioeconomic levels have less free facilities [61]. This problem is more severe in Brazil, where, despite the increased autonomy of sports bodies in recent years, it is the public funding that maintains the sports facilities [62]. In countries like Brazil, which often do not satisfactorily meet basic needs, the importance of sports may not be properly valued.

This study aimed to analyze factors related to sports participation in Brazil, based on a large national database. In multipurpose and wide-ranging surveys in terms of territorial extension, as is the case of PNAD, it is practically impossible to isolate errors that may influence the results. Such errors may arise from random fluctuations (sampling errors) or be non-probabilistic errors (other than sampling). Another possible limitation of the study is not considering the different socioeconomic and cultural realities of Brazil, a country that has continental dimensions. Future researches might clarify these disparities. Furthermore, the creation of a systematized longitudinal data collection would propitiate the understanding of the evolution of this process.

## 5. Conclusions

Concerning the sports profile of the population, the study was able to support some results already found in the literature, such as low sport participation rate, the decrease in participation rates with advancing age, the increase according to educational level, and male prevalence in the sports context. The results also indicated more difficulties for women in accessing sport, especially due to the lack of sports facilities or free access to them. For men, lack of time and health problems were the most common reasons given for not practicing sport. Women practice sports mainly due to medical recommendation, and men due to socialization, fun, and competition, especially through participation in soccer, demonstrating that Brazil is the country of soccer in the male context.

Internationally, this study can collaborate by reinforcing results and bringing new discoveries to this field of research, which is still expanding. The analysis of the impact of factors such as professional guidance, sports facilities, and participation in competitions in different sports can be considered a differential. Also, when discussing the reality of a country in Latin America with robust data, the study can facilitate the exchange of knowledge as well as the comparison with other regions of the world.

For Brazil, the findings may serve to support public policies for sports promotion. The results demonstrate that efforts are necessary to increase population awareness of the relevance of sports practice for the quality of life. Furthermore, sport should be encouraged as an important element for fun and socialization, being a fundamental alternative of leisure for the population. In addition, future actions may focus on providing access to sports facilities or promoting sports less dependent on facilities, such as cycling and running, targeting a greater female participation.

## Figures and Tables

**Table 1:** Sample distributed by gender, age groups and educational level.

Characteristics	People in the Sample
**TOTAL**	71,142
**Gender**	
Men	32,843
Women	38,299
**Age Groups**	
15 to 20 years old	6968
21 to 40 years old	28,355
41 to 59 years old	21,355
60 years old or older	14,464
**Educational Level**	
Unschooled	4609
Elementary School	28,629
High School	24,762
Undergraduate	12,382
Graduate	760

**Table 2:** Main results of the binary logistical regression—choice of practicing sports.

Source	Reference Level	*p*-Value *	Odds Ratio	Confidence Interval (95%)
Regression		0.000		
Age		0.000	0.98	(0.98;0.98)
Gender				
Men	Women	0.000	2.29	(2.20;2.37)
Educational Level				
Elementary	Unschooled	0.000	1.86	(1.65;2.08)
High School	Unschooled	0.000	2.94	(2.61;3.30)
Undergraduate	Unschooled	0.000	4.54	(4.03;5.11)
Graduate	Unschooled	0.000	5.91	(4.90;7.12)

* (α = 0.05).

**Table 3:** Main results of the nominal logistical regression—motives for not practicing sports.

Predictor	*p*-Value *	Odds Ratio	Confidence Interval (95%)
**Logit 1: (lack of time/lack of SF)**			
Age	0.000	1.01	(1.01;1.01)
Gender			
Women	0.000	0.65	(0.58;0.73)
Educational Level			
Graduate	0.001	3.12	(1.57;6.18)
**Logit 2: (health problems/lack of SF)**			
Age	0.000	1.08	(1.08;1.09)
Gender			
Women	0.000	0.65	(0.58;0.73)
Educational Level			
Graduate	0.065	0.50	(0.24;1.04)
**Logit 3: (does not like/lack of SF)**			
Age	0.000	1.02	(1.01;1.02)
Gender			
Women	0.000	0.74	(0.66;1.02)
Educational Level			
Graduate	0.309	0.70	(0.35;1.40)
**Logit 4: (lack of company/lack of SF)**			
Age	0.024	1.01	(1.00;1.01)
Gender			
Women	0.139	1.14	(0.96;1.37)
Educational Level			
Graduate	0.903	1.08	(0.31;3.77)
**Logit 5: (lack of money/lack of SF)**			
Age	0.014	1.01	(1.00;1.01)
Gender			
Women	0.001	1.34	(1.12;1.61)
Educational Level			
Graduate	0.821	0.87	(0.25;2.99)

* (α = 0.05). SF = sports facilities.

**Table 4:** Main results of the nominal logistic regression—motives for practicing sports.

Predictor	*p*-Value *	Odds Ratio	Confidence Interval (95%)
**Logit 1: (socialization/medical recommendation)**			
Age	0.000	0.92	(0.92; 0.93)
Gender			
Men	0.000	8.92	(7.05; 11.29)
Educational Level			
Graduate	0.060	0.32	(0.09; 1.05)
**Logit 2: (life quality/medical recommendation)**			
Age	0.000	0.97	(0.96; 0.97)
Gender			
Men	0.000	1.88	(1.66; 2.12)
Educational Level			
Graduate	0.000	3.38	(2.05; 5.56)
**Logit 3: (fun/ medical recommendation)**			
Age	0.000	0.92	(0.92; 0.93)
Gender			
Men	0.000	8.15	(7.14; 9.31)
Educational Level			
Graduate	0.224	0.69	(0.38; 1.25)
**Logit 4: (fitness/ medical recommendation)**			
Age	0.000	0.94	(0.94; 0.95)
Gender			
Men	0.000	2.78	(2.44; 3.16)
Educational Level			
Graduate	0.030	1.90	(1.07; 3.40)
**Logit 5: (competition/ medical recommendation)**			
Age	0.000	0.90	(0.90; 0.91)
Gender			
Men	0.000	9.38	(7.84; 11.23)
Educational Level			
Graduate	0.286	0.60	(0.23; 1.54)

* (α = 0.05).

**Table 5:** Main sports practiced (%).

Sport	Quantity	(%)
Soccer	4692	28.21
Walking	4480	26.94
Fitness Sports	1562	9.39
Futsal	1385	8.33
Others	908	5.46
Cycling	578	3.48
Combat/Martial Arts	525	3.16
Gymnastics	524	3.15
Bodybuilding/Weightlifting	461	2.77
Volleyball	356	2.14
Swimming/Diving	353	2.12
Athletics	260	1.56
Dance/Ballet	143	0.86
Small balls and rackets	88	0.53
Skateboarding/Skating	65	0.39
Water Sports	63	0.38
Basketball	57	0.34
Handball	41	0.25
Sport with animals	37	0.22
Adventure sports	19	0.11
Car sports	18	0.11
Cards and board games	15	0.09
**Total**		**100**

**Table 6:** Main results of the nominal logistical regression—main sport practiced.

Predictor	*p*-Value *	Odds Ratio	Confidence Interval (95%)
**Logit 1: (Volleyball/Soccer)**			
Age	0.555	1.00	(0.99; 1.01)
Gender			
Women	0.000	27.95	(21.40; 36.52)
Professional guidance			
Yes	0.000	4.19	(3.13; 5.60)
Participated in competition			
Yes	0.000	0.59	(0.44; 0.79)
Place of practice			
Free SF	0.000	2.36	(1.73; 3.22)
Paid SF	0.000	2.12	(1.54; 2.92)
**Logit 2: (Swimming and Diving/Soccer)**			
Age	0.000	1.09	(1.08; 1.10)
Gender			
Women	0.000	11.75	(8.80; 15.68)
Professional guidance			
Yes	0.000	40.33	(28.90; 56.28)
Participated in competition			
Yes	0.000	0.22	(0.15; 0.32)
Place of practice			
Free SF	0.319	1.28	(0.79; 2.06)
Paid SF	0.000	5.37	(3.57; 8.06)
**Logit 3: (Combat and Martial Arts/Soccer)**			
Age	0.000	1.03	(1.02; 1.04)
Gender			
Women	0.000	4.59	(3.52; 5.99)
Professional guidance			
Yes	0.000	139.27	(93.25; 208.00)
Participated in competition			
Yes	0.000	0.51	(0.40; 0.66)
Place of practice			
Free SF	0.323	1.24	(0.81; 1.88)
Paid SF	0.000	4.19	(2.89; 6.09)
**Logit 4: (Gymnastics/Soccer)**			
Age	0.000	1.09	(1.08; 1.10)
Gender			
Women	0.000	47.00	(35.04; 63.05)
Professional guidance			
Yes	0.000	81.34	(57.10; 115.88)
Participated in competition			
Yes	0.000	0.03	(0.01; 0.06)
Place of practice			
Free SF	0.005	0.59	(0.41; 0.86)
Paid SF	0.011	1.52	(1.10; 2.09)
**Logit 5: (Futsal/Soccer)**			
Age	0.000	0.97	(0.96; 0.98)
Gender			
Women	0.000	2.76	(2.19; 3.47)
Professional guidance			
Yes	0.654	1.04	(0.86; 1.26)
Participated in competition			
Yes	0.461	0.94	(0.81; 1.10)
Place of practice			
Free SF	0.000	6.43	(5.36; 7.73)
Paid SF	0.000	6.68	(5.57; 8.01)
**Logit 6: (Fitness sports/Soccer)**			
Age	0.000	1.06	(1.06; 1.07)
Gender			
Women	0.000	21.89	(17.58; 27.25)
Professional guidance			
Yes	0.000	62.19	(49.29; 78.45)
Participated in competition			
Yes	0.000	0.03	(0.02; 0.04)
Place of practice			
Free SF	0.000	0.55	(0.41; 0.74)
Paid SF	0.000	3.78	(2.98; 4.78)
**Logit 7: (Dance and Ballet/Soccer)**			
Age	0.000	1.07	(1.06; 1.08)
Gender			
Women	0.000	97.02	(55.73; 168.88)
Professional guidance			
Yes	0.000	43.47	(25.14; 75.17)
Participated in competition			
Yes	0.000	0.27	(0.16; 0.45)
Place of practice			
Free SF	0.311	0.75	(0.43; 1.31)
Paid SF	0.466	1.21	(0.72; 2.04)
**Logit 8: (Bodybuilding and Weightlifting/Soccer)**			
Age	0.000	1.05	(1.04; 1.06)
Gender			
Women	0.000	12.64	(9.63; 16.61)
Professional guidance			
Yes	0.000	64.56	(45.43; 91.75)
Participated in competition			
Yes	0.000	0.03	(0.02; 0.06)
Place of practice			
Free SF	0.239	0.69	(0.37; 1.28)
Paid SF	0.000	8.34	(5.22; 13.33)
**Logit 9: (Cycling/Soccer)**			
Age	0.000	1.06	(1.05; 1.07)
Gender			
Women	0.000	7.90	(6.20; 10.08)
Professional guidance			
Yes	0.000	3.28	(2.21; 4.87)
Participated in competition			
Yes	0.000	0.30	(0.22; 0.43)
Place of practice			
Free SF	0.000	0.03	(0.02; 0.06)
Paid SF	0.000	0.04	(0.02; 0.07)
**Logit 10: (Walking/Soccer)**			
Age	0.000	1.10	(1.09; 1.10)
Gender			
Women	0.000	48.37	(40.39; 57.91)
Professional guidance			
Yes	0.000	2.83	(2.23; 3.60)
Participated in competition			
Yes	0.000	0.04	(0.02; 0.05)
Place of practice			
Free SF	0.000	0.11	(0.09; 0.13)
Paid SF	0.000	0.10	(0.08; 0.13)
**Logit 11: (Athletics/Soccer)**			
Age	0.000	1.05	(1.04; 1.06)
Gender			
Women	0.000	8.59	(6.29; 11.72)
Professional guidance			
Yes	0.000	8.31	(5.83; 11.84)
Participated in competition			
Yes	0.001	1.62	(1.21; 2.18)
Place of practice			
Free SF	0.000	0.10	(0.06; 0.17)
Paid SF	0.000	0.13	(0.08; 0.20)

* (α = 0.05); SF = sport facilities.

## Appendix A

**Table A1:** Types of Sports—PNAD 2015

Adventure sport		Basketball	Bodybuilding/fitness	
Alpinism Bungee jumping Free climbing Free flight Hiking Mountain climbing	Parachuting Paragliding Rappel Slackline Tree climbing Zipline	Indoor Mini-basket Street	Bodybuilding Weight lifting Weight training	
**Cards and board**		**Car sports**	**Combat sports/martial arts**	
Card games Chess Dice Draughts		Car racing Enduro Karting Motocross Motorcycling powerboating Rally	Aikido Boxing Capoeira Full contact Jiu jitsu Judo Karate Kickboxing	Kung fu MMA Muay thai Sumo Taekwondo Tai chi chuan Wrestling
**Cycling**		**Dance/ballet**	**Fitness sports**	
Bmx Cycling street and road Cycling tour Mountain-bike		Ballroom Classic Contemporary Modern Sports	Aerobics Bikestretching Cardio fitness Functional training Gym Physical exercises Pilates	Spinning Stationary Step Treadmill Water aerobics Yoga
**Soccer**		**Futsal**	**Gymnastics**	
Beach Field Society		Futsal Indoor soccer	Artistic Rhythmic Trampoline	
**Handball**		**Skate/skating**	**Small balls and rackets**	
Beach Indoor		Rollerblading Skateboarding Skating	Badminton Beach tennis Billiards Bocha Bowling Court tennis Frescobol Golf	Padel Ping-pong Pool Racquetball Shuttlecook Squash Table tennis
**Sports with animals**		**Swimming/heels**	**Track and field**	
Dressage Equestrianism Horseback Polo Racing Riding Rodeo		Diving Synchronized swimming Swimming Water polo	Athletics Jumps Marathon Race walking Road running Throws Track races	
**Volleyball**		**Walking**	**Water sports**	
Beach Indoor Volleyball		Walking	Bodyboarding Canoeing Diving Fishing Kayaking Kitesurfing Rowing	Sailing Stand-up paddle Surfing Underwater fishing Waterskiing Windsurfing
